# A Symbiotic Fungus *Sistotrema* Benefits Blueberry Rejuvenation and Abiotic Stress Tolerance

**DOI:** 10.3390/jof9070779

**Published:** 2023-07-24

**Authors:** Yu Ye, Xufang Zhan, Kai Wang, Jingya Zhong, Fanglei Liao, Wenrong Chen, Weidong Guo

**Affiliations:** 1 College of Life Sciences, Zhejiang Normal University, Jinhua 321004, China; yeyu87@zjnu.edu.cn (Y.Y.); zxfsophia@163.com (X.Z.); surprise_sang@zjnu.edu.cn (J.Z.); gwd@zjnu.cn (W.G.); 2 School of Marine Sciences, Ningbo University, Ningbo 315211, China; wangkai@nbu.edu.cn; 3 Collaborative Innovation Center for Zhejiang Marine High-Efficiency and Healthy Aquaculture, Ningbo 315211, China; 4 Zhejiang Provincial Key Laboratory of Biotechnology on Specialty Economic Plants, Zhejiang Normal University, Jinhua 321004, China

**Keywords:** blueberry, fungal reinoculation, rhizosphere fungi analysis, growth-promoting effect, optimization of soil environment

## Abstract

Blueberry (*Vaccinium* spp.) rhizosphere microorganisms can significantly increase the absorption area and improve the efficiency of rhizospheric nutrient uptake. However, there has been little research on blueberry rhizosphere microorganisms, especially those that can complement root function deficiency. In this study, we analyzed the rhizosphere fungi of ‘O’Neal,’ ‘Sharpblue,’ and ‘Premier’ blueberry cultivars and found that ‘Premier’ blueberries showed strong growth potential and relatively high root regulation ability. The dominant symbiotic fungus *Sistotrema* was correlated with the strong growth of ‘Premier’ and was directionally screened and isolated based on conserved gene structures and COG function analysis. This fungus was reinoculated onto the roots of ‘Gulfcoast’ and ‘Star’ blueberry cultivars. *Sistotrema* promoted the growth of blueberries and improved their ability to resist stress and grow under adverse conditions, as indicated by maintained or increased chlorophyll content under such conditions. Further analyses showed that *Sistotrema* has certain functional characteristics such as the ability to dissolve iron in its insoluble form and then release it, to fix nitrogen, and to inhibit nitrification in soil. Thus, it effectively doubled the soil nitrogen content and increased the soluble iron content in soil by 50%. This investigation indicates *sistotrema* inoculation as an approach to increase blueberry stress tolerance and complete their root nutrition deficiency.

## 1. Introduction

The rhizosphere is the narrow zone of soil around a plant’s roots that is affected by root exudates, and it is the primary location where plants interact with soil and root symbiotic fungi [1]. Rhizosphere microorganisms play a crucial role in increasing crop yield, controlling diseases and insect pests, and preventing problems associated with continuous cropping [2]. Some microorganisms can improve plants’ resistance to stresses through beneficial rhizosphere interactions [3], and some soil microorganisms can also protect plant roots from fungal pathogens [4]. Symbiotic microorganisms in the rhizosphere such as mycorrhizal fungi and rhizobia can indirectly improve plants’ nutritional status by increasing the absorption area of plant roots through mycelia [5]. They can also transform nutrients that are not directly usable by plants into free or available forms in the soil, which can effectively increase nutrient availability to plants for growth [6]. In addition, microorganisms that fix nitrogen can effectively inhibit nitrification–denitrification and weaken nitrification in soil, especially under drought conditions, thereby preventing an increase in soil pH [7]. Inorganic nitrogen, NH_4_–N, and NO_3_–N resulting from microbial nitrogen fixation and organic matter degradation can be directly absorbed and utilized by plants [8]. In turn, plant root exudates can provide nutrients to symbiotic microorganisms [9] and stimulate microbial degradation in the rhizosphere [10]. Moreover, these exudates mediate a range of rhizosphere interactions (plant–microbial and microbial–microbial), and affect the distribution of the soil microbial community [11]. The diversity of soil microbes is remarkably high, with approximately 10^7^ prokaryotes per gram of soil. However, it is important to note that only 0.1% to 10% of these microorganisms can be cultured [12], leaving a vast number of microorganisms unrecognized and unexploited.

Blueberry is a shallow-rooted plant with an underdeveloped root system and no taproot. As a result, it has a limited ability to access nutrients and water from deep soil, making it vulnerable to drought stress. Moreover, blueberry roots are sensitive to changes in pH, and high pH can lead to leaf chlorosis and stunted growth [13]. An analysis of the microbial community in blueberry rhizosphere soil revealed the positive role of mycorrhizal fungi in enhancing plant growth and improving the quality and yield of the crop [14]. Inoculation with mycorrhizal fungi was found to effectively enhance blueberry growth, increase the soluble sugars content in the plants [15], and increase the concentration of inorganic nitrogen in soil [8]. Therefore, determining the effect of reinoculation with symbiotic fungi in the blueberry root system is useful from both research and production perspectives. However, there is a relative lack of research on functional fungi in the blueberry root system. Most of the strains used in previous inoculation studies were commercialized or isolated from different plant species [16], resulting in a weak inoculation effect and sometimes the inhibition of plant growth. Heterogenous strains lack plant specificity and often have a low inoculation rate [17]. In the small number of studies on functional microorganisms associated with blueberry, the identification, isolation, and inoculation of microorganisms in blueberry roots have mainly relied on traditional methods. These involve isolating symbiotic strains from blueberries and then verifying their characteristics through the determination of various physical and chemical indices, and then determining the function of strains and their symbiotic effect. However, these methods are untargeted and often require a significant amount of resources and labor [18]. While several studies have reported on the metagenomes of microbes in the blueberry rhizosphere, they have generally compared different species or varieties of blueberry, and relevant microbial diversity results have seldom been used in downstream production [19]. Therefore, it is crucial to rapidly and accurately screen and isolate beneficial symbiotic fungi from blueberry and construct a stable microflora in the rhizosphere of blueberry to achieve the best effects in terms of growth and stress resistance.

In this study, we analyzed the diversity and functions of soil fungi communities of blueberry plants of different varieties and in different growth states, and identified the fungus *Sistotrema* with a potential growth-promoting effect. We conducted COG functional analyses to predict its functions, and found that it has the capacity to improve the nutrient status of soil. Then, we used the motif sequence obtained by MEME comparison to directionally screen *Sistotrema* colonies. To test its efficacy, isolated *Sistotrema* was inoculated onto blueberry plants, and then the plants were subjected to a nutrient-deficiency- and drought-stress treatment. The plants inoculated with *Sistotrema* showed improved stress resistance under these conditions. In addition, weak and pruned plants inoculated with *Sistotrema* showed better recovery and resprouting under normal growth conditions.

## 2. Materials and Methods

### 2.1. Plant Materials, Soil Sampling, Reinoculation, and Experimental Design

The blueberry varieties ‘O’Neal’ (O), ‘Sharpblue’ (X), and ‘Premier’ (J) were used in this study; these varieties represent the three growth states of weak, medium, and strong growth in central Zhejiang, China. Soil samples were taken from the rhizosphere and nonrhizosphere of 5-year-old plants. The sample codes are as follows: rhizosphere soil collected from vigorous plants (JG, XG, O) and weak plants (JB, XB) and nonrhizosphere soil (JGK, JBK, XGK, XBK, OK) (Table 1). The sampler was disinfected between each sample, and sampling points of nonrhizosphere soil were at least 1 m apart. Each group had five replicates.

For the reinoculation tests, we used 3-year-old plants of the ‘Star’ blueberry and 1-year-old plants of the ‘Gulfcoast’ blueberry. ‘Star’ seedlings show poor growth, and ‘Gulfcoast’ is particularly sensitive to drought stress. The plant materials were transplanted from the original cultivated soil into a mixed substrate of coconut bran, vermiculite, and mica (100:1:1) after cleaning the roots. To highlight any effects and shorten the screening time, we established the following stress conditions: water shortage (watering every 5 d); nutrient shortage (no exogenous nutrients added during cultivation); and high temperature (noon temperature maintained at 35–40 °C). The plants were cultivated for 50 d under these conditions.

Further reinoculation tests were conducted using 3-year-old dying ‘Star’ and ‘Jewel’ blueberry seedlings (both cultivars showed a high mortality rate during seedling growth) and stunted ‘Star’ and ‘Jewel’ blueberry seedlings, and then rejuvenation and budding were evaluated after inoculation. These plants were not transplanted (Table 2).

Each group had six biological replicates. The plant materials were obtained from the germplasm resource nursery of our research group. Under normal cultivation conditions, the plants were irrigated with 1× Hoagland’s soil nutrient solution (Supplementary Table S1) every 2 days (approximately 150 mL per plant).

### 2.2. Soil DNA Extraction

Improved direct and indirect extraction methods [20] were used to obtain DNA from the studied soil, which had a high humus and metal ion content. These improved methods yielded DNA from soil with sufficient quality for sequencing. The soil samples were washed with phosphoric acid buffer (pH 6.5) and centrifuged at 10,000× *g* for 5 min, and then the supernatant was removed. This was repeated 1–2 times. The soil pellet was resuspended in phosphoric acid buffer solution (pH 6.5) and placed in an ice bath. The larger particles, sand, and small stones settled to the bottom, leaving an upper turbid soil solution. The turbid solution was centrifuged, and then the pellet was subjected to repeated freeze–thawing with liquid nitrogen and boiling water to split the cells. This process was repeated 3–5 times. Then, lysozyme and proteinase K were added to dissolve the cell walls and remove proteins. The mixture was treated with CTAB at 65 °C for 1 h and then centrifuged. The supernatant was mixed with an equal volume of chloroform: isoamyl alcohol (24:1), then tris-buffered phenol (pH 7.8) was added to remove impurities such as proteins, polysaccharides, and phenols, and the mixture was centrifuged. The supernatant was collected and the previous step was repeated. DNA was precipitated from the upper aqueous phase by adding an equal volume of anhydrous ethanol and 0.1 volume of NaAc (pH 5.2). The mixture was centrifuged, and the supernatant was discarded. The precipitate was washed twice with anhydrous ethanol and then air-dried before 30 μL of ddH_2_O was added to dissolve the DNA. Finally, the concentration of DNA was determined using a NanoDrop 2000 nucleic acid analyzer (Nanodrop Technologies, Wilmington, DE, USA). The quality of DNA was detected by gel electrophoresis, and its integrity was verified by PCR.

### 2.3. ITS Gene Sequencing, Data Processing, and Taxonomic Assignments

The ITS1 regions of fungal genomes were amplified using universal primers (Supplementary Table S2). The PCR products were sequenced by Nevogene Co., Ltd. (Beijing, China). All DNA sequence analyses were performed using the QIIME pipeline (Version 1.9.1) [21]. the Unite database [22] was used for fungal analysis. The programs USEARCH (version 10) and VSEARCH (Version 2.10.4) were used to generate the OTU table and annotate species. We used USEARCH-UNOISE3-zOTUs to obtain OTU data, and the results were consistent with the amplicon-sequencing variant (ASV) data derived from DAD2. Because USEARCH was used in this study, we retained the output naming convention as OTU. An OTU table was constructed to compare alpha diversity (Chao1 index, Shannon’s index) and beta diversity (Bray–Curtis dissimilarity matrix and weighted UniFrac distance) within and among groups [23].

### 2.4. Functional Predictions Based on ITS Regions

FAPROTAX was used to predict the metabolic functions of the bacterial community [24]. Functional predictions based on fungal ITS data were conducted using the Python script of FUNGuild [25].

### 2.5. Data Visualization and Analysis

The differences in rhizosphere fungi groups among samples were analyzed by VennDiagram (R package) [26] and visualized in the form of Venn petal diagrams. Comparisons between rhizosphere and nonrhizosphere soil and among all sample groups were made using UpSetR (R package) [27].

Co-occurrence network analyses were performed to assess correlations among rhizosphere fungi. The *p* value was used for data correction (psych, R package), and igraph (R package) was used to calculate and visualize correlations [28]. In this analysis, the labels of nontarget clusters were removed during the clustering of bacterial groups. The proportion of pathogenic bacteria was calculated, and comparisons were made between rhizosphere and nonrhizosphere soil samples as well as among different rhizospheres. These analyses allowed us to link the health status of blueberry roots with the functions of fungal communities, as determined by the functional analysis of the fungal ITS macrogenome. The number of fungi and the number of pathogenic bacteria were important biological indicators of the health of the soil environment [29].

### 2.6. COG Functional Annotation

A COG (clusters of orthologous groups) analysis [30] of the reinoculated fungi genome was performed using EggNOG script, and Diamond was used as the comparison algorithm. The EggNOG fungus database was used in these analyses. The results were plotted and analyzed using ggplot2 (R package).

### 2.7. Directional Isolation of Fungal Strains and Reinoculation

A MEME comparison [31] was carried out for *Sistotrema*, the most abundant symbiotic fungi in the rhizosphere of the ‘Premier’ blueberry. The genomes of 71 *Sistotrema* species (obtained from the Unite database) from around the world were clustered to identify motif sequences. Appropriate fragments were selected as specific primers for colony PCR to screen the target strains. Fungal strains were isolated from the rhizosphere soil of blueberry on PDA medium (containing streptomycin), and then selected strains were suspended in ddH_2_O. Then, the fungus solution was used as a template to conduct directed colony PCR screening using the specific screening primers (Supplementary Table S3) obtained in the MEME analysis. Each isolated colony was sent to a commercial company for sequencing using the sequencing primers ITS1F/R (Supplementary Table S2). A strain that was successfully sequenced and verified was selected as the target strain for isolation and purification.

After verification by sequencing, a 200 µL aliquot of the target fungal strain was cultured on PDA medium in the dark for 2–3 days at 28 °C. When the medium was completely covered with mycelia, small pieces (1 × 1 × 0.5 cm) were removed to inoculate the rhizosphere of plants, with three to four pieces per seedling.

The target fungal strain was inoculated onto the roots of 1-year-old ‘Gulfcoast’ blueberry plants. The plants were cultivated for 50 days under drought, nutrient-deficiency, and high-temperature conditions (see Section 2.1). Measurements and observations were conducted every 10 days. The first 10 days represented the stage of fungus propagation and plant growth adaptation (beginning at day 0).

The target fungal strain was also inoculated into the rhizosphere of 3-year-old ‘Star’ and ‘Jewel’ blueberry plants growing under normal conditions. Each 3-year-old dying and pruned ‘Star’ and ‘Jewel’ blueberry plant was inoculated with three to four mycelial blocks to evaluate the effect of reinoculation on rejuvenation and sprouting. The blueberry plants were observed for 40 days after fungal inoculation.

### 2.8. Determination of Physiological Indexes

Chlorophyll was extracted from the leaves of ‘Gulfcoast’ blueberry in 95% *v*/*v* ethanol, and then the absorbance of the extract was measured at 665 nm, 649 nm, and 470 nm using an ultraviolet spectrophotometer (Agilent Technology Co., Ltd., Shanghai, China). The chlorophyll content was calculated as described by Mansouri and Salari [32].

Root activity was measured using the TTC (2,3,5-triphenyl tetrazolium chloride) method [33]. Fresh blueberry roots (0.5 g) were soaked in TTC buffer solution (5 mL pH 7.0 phosphate buffer and 5 mL 0.4% TTC) at 37 °C with shaking at 180 rpm for 2 h in the dark. Then, 1 mL 1 M H_2_SO_4_ was added to terminate the reaction. Triphenylmethylene was extracted from the soaked root samples with ethyl acetate. The volume of the extract was completed to 10 mL and the absorbance value was measured at 485 nm. A standard sample was prepared with triphenyl hydrazone–ethyl acetate.

The anthrone–sulfuric acid assay method was used to determine the total sugars content in plant samples [34]. Powdered samples of roots, stems, and leaves (0.1 g) that had been passed through a 100-mesh sieve were extracted with ddH_2_O (10 mL) at 90 °C for 4 h. After cooling to room temperature, the mixture was centrifuged. The supernatant was diluted before adding anthrone–sulfuric acid solution, and then the mixture was heated in a boiling water bath for 10 min before cooling to room temperature. After standing for 10 min, the absorbance value was measured at 627 nm. When polysaccharides are hydrolyzed to monosaccharides, one glycosidic bond consumes one H_2_O molecule, so it is necessary to multiply by a correction coefficient of 0.9 when calculating the total sugar content.

Reducing sugars in plant samples were quantified using the 3,5-dinitrosalicylic acid (DNS) method [35]. The DNS reaction solution (Supplementary Table S4) was stored in a brown bottle and remained stable for 7 days at 4 °C in the dark. The extract solution was diluted and DNS reaction solution was added, then the mixture was placed in a boiling water bath for 5 min before cooling to room temperature. After standing for 10 min to stabilize the color of the reaction solution, the absorbance value was measured at 540 nm.

Iron (Fe) was extracted from rhizosphere soil samples using diethylenetriaminepentaacetic acid (DTPA) [36]. Each rhizosphere soil sample (1.0 g) was extracted with DTPA (10 mL) for 2 h with shaking at 180 rpm [37]. The mixture was filtered through a 0.22 μm filter, the filtrate was diluted 2000 times, and then the absorbance value of the diluted filtrate at 248.33 nm was determined using a graphite atomic absorption spectrometer. The measurement was repeated three times with DTPA as the blank.

For analyses of soil nitrogen content, the rhizosphere soil samples (1.0 g) were extracted with ddH_2_O for 2 h with shaking at 180 rpm, and then the extract was filtered through a 0.22 μm filter membrane. The filtrate was analyzed to determine the NH_4_–N and NO_3_-N contents using Nessler’s reagent [38] and a spectrophotometric method [39], respectively. The total soluble inorganic nitrogen content in soil samples was the sum of soluble ammonium nitrogen and nitrate nitrogen [8].

### 2.9. Statistical Analysis

All the data in figures and tables are means ± standard deviations (SD) from at least three independent experimental replicates per sample. Data were analyzed using dplyr (R package). The statistical significance of differences between means was analyzed using tidyverse (R package) at the 0.05 probability level. Graphs were generated using ggplot2.

## 3. Results

### 3.1. Diversity Analysis of Fungi in Rhizosphere and Non-Rhizosphere Soils of Different Blueberry Cultivars

**Figure 1:**
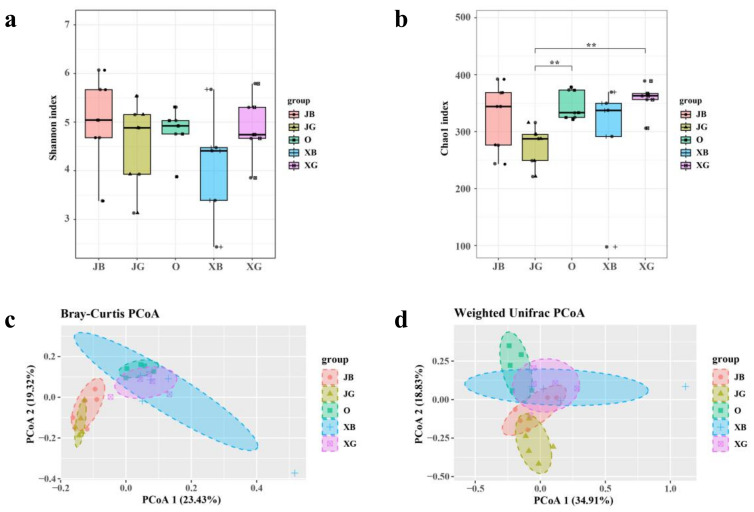
Diversity analysis of fungi in rhizosphere and nonrhizosphere soils of different blueberry cultivars. Alpha diversity analysis of (**a**) Shannon’s index and (**b**) Chao1 index. Beta diversity analysis of (**c**) fungi Bray–Curtis dissimilarity of fungal communities and (**d**) weighted UniFrac dissimilarity of fungal communities. Note: sample codes for rhizosphere soil samples: JG, flourishing ‘Premier’ plants; JB, weak ‘Premier’ plants; O, ‘O’Neal’ plants; XG, flourishing ‘Sharpblue’ plants; XB, weak ‘Sharpblue’ plants. *p* < 0.01 **.

The rhizosphere and nonrhizosphere soil samples from ‘Premier,’ ‘Sharpblue,’ and ‘O’ Neal’ were analyzed to detect the alpha diversity (Chao1 and Shannon’s index) of their fungal communities. The results showed that there were more species of fungi in the rhizosphere soil of ‘O’Neal’ (O), and fewer in the rhizosphere soil of ‘Premier’ (JG, JB). Previously, we found that ‘Premier’ blueberry has stronger environmental adaptability, while ‘O’Neal’ blueberry has poorer environmental adaptability [40]. Because plant roots themselves affect soil microbial structure [29], we speculated that ‘Premier’ blueberry (JG, JB) roots may have a stronger ability than those of other cultivars to support microbes, and may enrich beneficial rhizosphere microorganisms (Figure 1a,b).

We conducted beta diversity analyses of fungi in the rhizosphere and nonrhizosphere soil of ‘Premier,’ ‘Sharpblue,’ and ‘O’Neal.’ The results of the Bray–Curtis (Figure 1c) and weighted UniFrac (Figure 1d) analyses revealed that there were differences in fungal community composition among groups, with less overlap among groups, and the groups were uniformly distributed on the plot, which also revealed substantial differences in the fungal community of ‘Premier’ compared with those of ‘Sharpblue’ and ‘O’Neal.’

### 3.2. Functional Prediction Based on Fungal ITS Sequences and Estimation of Blueberry Root Health Status

**Figure 2:**
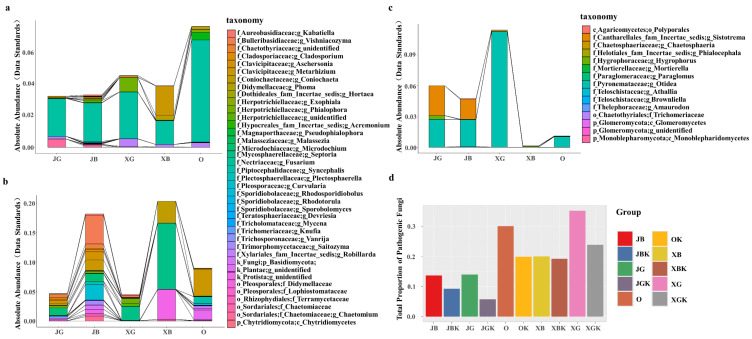
Root fungal functional prediction and estimation of blueberry root health status of different blueberry varieties. (**a**) Predicted pathogenic fungi. (**b**) Predicted saprophytic fungi. (**c**) Symbiotic and saprophytic–symbiotic fungi and their abundance as predicted using FUNGuild. (**d**) Estimation of environmental health of rhizosphere and nonrhizosphere soils of different blueberry varieties. Sample codes for rhizosphere soil samples: JG, flourishing ‘Premier’ plants; JB, weak ‘Premier’ plants; O, ‘O’Neal’ plants; XG, flourishing ‘Sharpblue’ plants; and XB, weak ‘Sharpblue’ plants. Absolute abundance was calculated after data standardization.

Among all the blueberry varieties, ‘Premier’ had the smallest number of pathogenic fungi in the rhizosphere (JG, JB). The smallest number of pathogenic bacteria in the rhizosphere was in flourishing ‘Premier’ plants (JG), followed by weak ‘Premier’ plants (JB), and then ‘Sharpblue’ (XG, XB) and ‘O’Neal’ plants (Figure 2a). The abundance of saprophytic fungi in rhizosphere soil was significantly lower in flourishing plants of ‘Premier’ and ‘Sharpblue’ (JG, XG) than in their weak counterparts (JB, XB), while the abundance of saprophytic fungi in rhizosphere soil was significantly higher in ‘O’Neal’ than in the flourishing plants of ‘Premier’ and ‘Sharpblue’ (JG, XG) (Figure 2b), indicating that the root systems of the weakly growing plants had rotted. The relative abundance of symbiotic fungi in rhizosphere soil was higher in the flourishing plants of ‘Premier’ (JG) than in its weakly growing counterpart (JB), but symbiotic fungi were not detected in the rhizosphere soil of the other varieties (XG, XB, O) (Figure 2c). This result indicated that there was a good correlation between symbiotic fungi and strong blueberry plant growth.

The pathogenicity of the root soil of each variety of blueberry was determined according to the proportion of pathotrophic fungi in the total fungi in the sample. There were more pathogenic bacteria in rhizosphere soils (JG, XG, O, JB, and XB) than in nonrhizosphere soils (JGK, JBK, XGK, XBK, and OK). The difference in the number of pathotrophic strains between rhizosphere and nonrhizosphere soils was smaller for the weaker plants than for the flourishing plants (Figure 2d). On the whole, flourishing seedlings (JG, XG) had a stronger inhibitory effect than weak seedlings (JB, XB) on pathotrophic fungi in soil. The root microecology of blueberry ‘Premier’ (JG, JB) was the healthiest, followed by ‘O’Neal.’ The root microecology of flourishing ‘Sharpblue’ (XG) was the least healthy.

### 3.3. Rhizosphere Fungal Co-Occurrence Networks and Differences in Rhizosphere Fungi among Various Blueberry Cultivars

**Figure 3:**
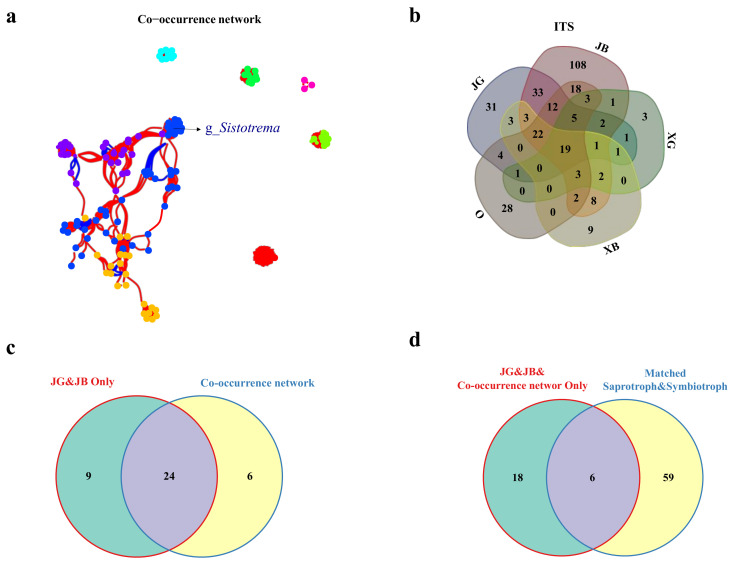
Analysis of interaction network and differences in microorganisms in the rhizosphere among different blueberry cultivars. (**a**) Interaction network of different blueberry rhizosphere fungi (red edge between two nodes shows a positive correlation, and blue edge shows a negative correlation; for simplicity, only the name of the fungus we focused on is shown). (**b**) Difference analysis of fungi in different blueberry rhizospheres. (**c**) Difference analysis of fungi unique to flourishing ‘Premier’ plants (JG) and weak ‘Premier’ plants (JB) and fungi with microbial network correlation. (**d**) Difference analysis between ‘Premier’ (JG and JB) symbiotic fungi associated with microbial network and ‘Premier’ (JG and JB) saprophytic and symbiotic fungi. Sample codes for rhizosphere soil samples: JG, flourishing ‘Premier’ plants; JB, weak ‘Premier’ plants; O, ‘O’Neal’ plants; XG, flourishing ‘Sharpblue’ plants; and XB, weak ‘Sharpblue’ plants. Sample codes for nonrhizosphere soil samples: JGK, JBK, OK, XGK, XBK.

The results of the rhizosphere fungal co-occurrence network analysis showed that the most correlated symbiotic fungal community was the blue community (Figure 3a; for simplicity, only the name of the fungus we focused on is shown). A total of 30 fungi (Details shown in Supplementary Table S5) showed significant correlations with each other, i.e., they were likely to co-occur in a community (Figure 3a). This result served as the basis for follow-up difference analysis.

As shown in the Venn diagrams (Figure 3c), there were significant differences in fungal genera. In particular, there were more fungi endemic to ‘Premier’ (JG, 31 genera; JB, 108 genera; and JG and JB, 33 genera) than to other groups (Figure 3b).

Based on analyses of fungal ITS sequences and the rhizosphere fungal co-occurrence network, it was found that among the 33 genera of fungi unique to ‘Premier’ (Figure 3b), 24 genera of fungi were correlated with each other (i.e., were likely to co-occur) (Figure 3c), of which 6 had symbiotic abilities (Figure 3d).

**Figure 4:**
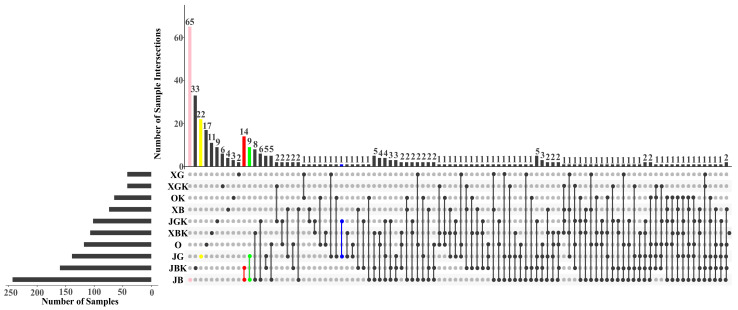
Difference analysis of fungi (including rhizosphere and non-rhizosphere soils). The UpSet plot is analogous to a Venn diagram. Vertical bars show genera shared among samples. Horizontal bars represent the total number of genera detected in each sample. Sample codes for rhizosphere soil samples: JG, flourishing ‘Premier’ plants; JB, weak ‘Premier’ plants; O, ‘O’Neal’ plants; XG, flourishing ‘Sharpblue’ plants; and XB, weak ‘Sharpblue’ plants. Sample codes for non-rhizosphere soil samples: JGK, JBK, OK, XGK, XBK.

The UpSet plot revealed that the rhizosphere of weak ‘Premier’ plants (JB) had the highest number of endemic fungi, double that in its nonrhizosphere soil (JBK), with 14 fungal genera shared between JB and JBK. The number of endemic fungi in the rhizosphere of flourishing ‘Premier’ plants (JG) was 2.5 times higher than that in its nonrhizosphere soil (JGK), but only one fungal genus was shared between these two groups. Thus, the difference in fungal communities between rhizosphere and nonrhizosphere soil of ‘Premier’ was smaller for the flourishing plants than for the weak plants; furthermore, it was smaller for ‘Premier’ than for ‘Sharpblue’ and ‘O’Neal.’ Therefore, we speculated that the root exudates of ‘Premier’ had stronger selective capacity than those of ‘Sharpblue’ and ‘O’Neal’ blueberry, and its effect extended to nonrhizosphere soil (comparing X and O nonrhizosphere soil in the same region) (Figure 4).

### 3.4. Enrichment Analysis of COG Functional Classes of Reinoculated Fungi

**Figure 5:**
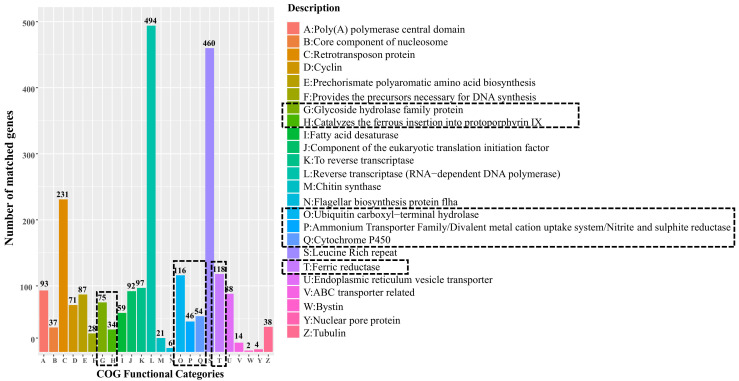
Enrichment analysis of COG functional categories of reinoculated *Sistotrema.*

The COG functional distribution of the genome of *Sistotrema* was analyzed. The results showed that the fungus had six main growth-promoting functions, namely: (1) G: glycoside hydrolase family protein; (2) H: catalysis of ferrous insertion into protoporphyrin IX; (3) O: ubiquitin carboxyl–terminal hydrolase; (4) P: ammonium transporter family/divalent metal cation uptake system/nitrite and sulfite reductase; (5) Q: cytochrome P450; and (6) T: ferric reductase. Among them, the most abundant functions were (O) ubiquitin carboxyl–terminal hydrolase and (T) ferric reductase, followed by (G) glycoside hydrolase family proteins (Figure 5).

These results show that *Sistotrema* has strong Fe reductase, glycoside hydrolysis, and anti-ubiquitin activities, which are complementary to the root characteristics of blueberry plants and have the potential to increase plant yield and/or growth. In addition, *Sistotrema* accounted for the largest proportion of symbiotic fungi unique to ‘Premier’ (Figure 3c), so it was relatively easy to isolate. Finally, we speculated that the fungus of *Sistotrema* was the most suitable for inoculation. The results of COG function analysis were further used as the basis for the determination of follow-up indicators.

### 3.5. Effects of Reinoculation with Symbiotic Fungi on Chlorophyll Content and Root Activity of Blueberry under Drought and Deficiency Stress

**Figure 6:**
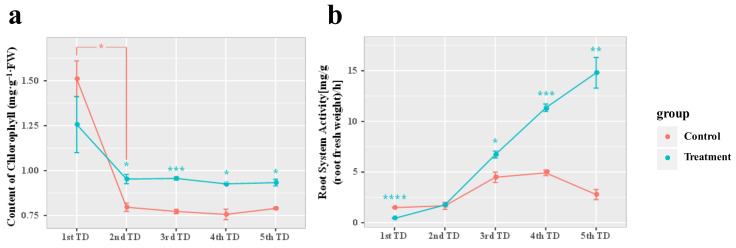
Effects of drought- and nutrient-deficiency-stress treatment (50 d) on ‘Gulfcoast’ blueberry reinoculated with endophytic fungi, compared with control group. Figure shows changes in (**a**) chlorophyll content and (**b**) root system activity. Asterisks indicate significant differences (*p* < 0.05 *, *p* < 0.01 **, *p* < 0.005 ***, *p* < 0.001 ****). Plants in the control were inoculated with PDA without microorganisms.

The chlorophyll content of ‘Gulfcoast’ blueberry reinoculated with *Sistotrema* symbiotic fungi decreased significantly by day 20 (2nd TD, where TD is defined as 10 days) of growth under nutrient-deficiency and drought stress. On day 20 (2nd TD) and later (3rd TD–5th TD) during the stress treatment, chlorophyll degradation was alleviated in the plants inoculated with *Sistotrema* symbiotic fungi compared with that in the control (Figure 6a).

On day 10 (1st TD) of the nutrient-deficiency- and drought-stress treatment, the root activity of ‘Gulfcoast’ blueberry was significantly lower in the group reinoculated with *Sistotrema* symbiotic fungi than in the control group (*p* < 0.01). On day 20 (2nd TD), the root activity had recovered, and in the following stage (3rd TD–5th TD), the root activity was significantly higher in the group reinoculated with *Sistotrema* symbiotic fungi than in the control group (*p* < 0.05). On day 50 (5th TD), the root activity of blueberry plants in the control group had decreased, while that of the blueberry plants reinoculated with *Sistotrema* symbiotic fungi was six times that on day 0 (Figure 6b).

### 3.6. Effects of Symbiotic Fungi Reinoculation on Carbohydrate Synthesis in Blueberry under Drought and Nutrient-Deficiency Stress

**Figure 7:**
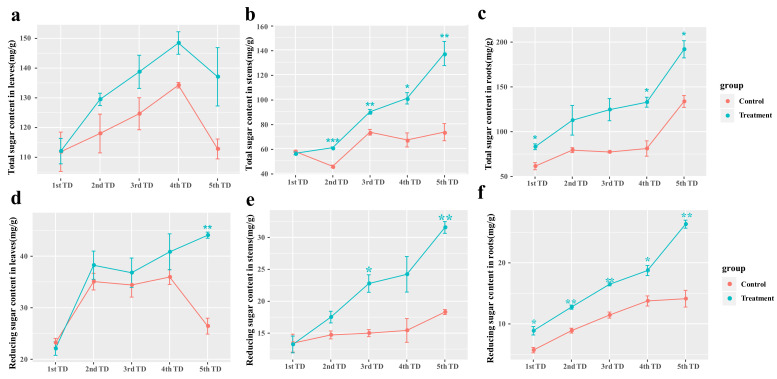
Effects of drought- and nutrient-deficiency-stress treatment (50 d) on ‘Gulfcoast’ blueberry reinoculated with endophytic fungi, compared with control group. Figure shows changes in total sugar content in (**a**) leaves, (**b**) stems, and (**c**) roots; and reducing sugars content in (**d**) leaves, (**e**) stems, and (**f**) roots. Asterisks indicate significant differences (*p* < 0.05 *, *p* < 0.01 **, *p* < 0.005 ***). Plants in the control were inoculated with PDA without microorganisms.

After day 10 (2nd TD–5th TD) of the nutrient-deficiency- and drought-stress treatment of ‘Gulfcoast’ blueberry, the total sugars content in leaves was higher in the group reinoculated with *Sistotrema* than in the control group, but there was no significant difference in each time period. In the stem, the transported volume of total sugars was significantly higher in the group reinoculated with *Sistotrema* than in the control group (*p* < 0.05) from the 2nd TD to the 5th TD. In the control group, there was no increase in the transported volume of sugars from the 3rd TD to the 5th TD. Sugars gradually accumulated in the roots throughout the treatment time (1st TD–5th TD). The total sugars content in roots was significantly higher in the group reinoculated with *Sistotrema* than in the control group (*p* < 0.05) at the later stage of the stress treatment (4th TD and 5th TD) (Figure 7a–c).

Under nutrient-deficiency and drought stress, reducing sugars accumulated to higher levels in the reinoculated group than in the control group at the later stage of treatment (5th TD). In the stem, the reducing sugars content did not differ significantly between the control group and the reinoculated group at each treatment stage (1st TD–5th TD), but reducing sugars synthesis was significantly higher in the reinoculated treatment group than in the control group (*p* < 0.05), especially at the later stage of stress treatment (3rd TD–5th TD). Reducing sugars accumulated in the roots to significantly higher levels in the reinoculated treatment group than in the control group on day 50 (5th TD) (Figure 7d–f).

### 3.7. Effects of Symbiotic Fungi Reinoculation on Soluble Iron and Nitrogen Contents in Rhizosphere Soil

**Figure 8:**
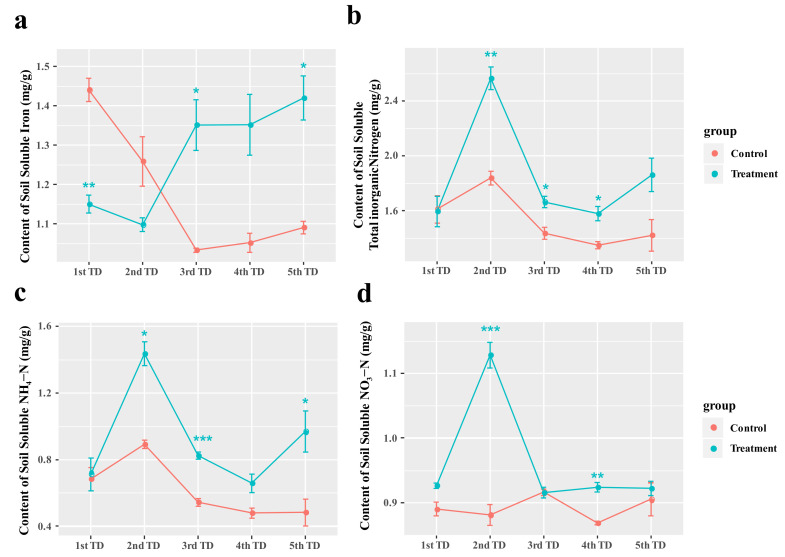
Effects of drought- and nutrient-deficiency-stress treatment (50 d) on ‘Gulfcoast’ blueberry reinoculated with endophytic fungi, compared with control group. Figure shows changes in (**a**) soil soluble-iron content and soil soluble-nitrogen content including (**b**) nitrate nitrogen (NO_3_–N), (**c**) ammonium nitrogen (NH_4_–N), and (**d**) total nitrogen. Asterisks indicate significant differences (*p* < 0.05 *, *p* < 0.01 **, *p* < 0.005 ***). Plants in the control were inoculated with PDA without microorganisms.

Under nutrient-deficient- and drought-stress conditions, reinoculation with *Sistotrema* significantly affected the soluble iron (Fe) content in the rhizosphere soil of ‘Gulfcoast’ blueberry plants (Figure 8a). The soluble Fe content in blueberry rhizosphere soil was significantly lower in the group reinoculated with *Sistotrema* than in the control group at day 20, but it increased significantly (*p* < 0.05) during the 2nd to 3rd TD in the group reinoculated with *Sistotrema* and remained stable at approximately 1.4 mg/g soil in the later stage (4th TD–5th TD). In the control, the soluble Fe in soil decreased continuously during the stress treatment and stabilized at approximately 0.5 mg/g soil. Blueberry plants consume approximately 0.2 mg/g free Fe every 10 days under stress.

We determined the contents of total inorganic nitrogen, NH_4_-N, and NO_3_-N in the rhizosphere soil of ‘Gulfcoast’ blueberry under nutrient-deficiency and drought stress, with and without reinoculation with *Sistotrema* (Figure 8b–d). The contents of NO_3_-N, NH_4_-N, and total inorganic nitrogen in rhizosphere soil peaked on day 20 (2nd TD), and the contents of NH4-N and total inorganic nitrogen also increased significantly in the later stage (4th and 5th TD) of treatment (*p* < 0.05). The nitrogen content in soil was significantly higher in the reinoculated group than in the control group at all stages of the stress treatment (1st TD–5th TD). At the later stages of stress treatment (2nd–5th TD), nitrification decreased significantly (*p* < 0.005), and the NO_3_-N content in soil remained stable at approximately 0.9 mg/g soil, which may represent the amount of NO_3_-N fixed by soil and not used by plants. The change in NH4-N content in soil during the 2nd and 3rd TD also indicated that the first 20 days comprised the stage of fungal reproduction and infection.

### 3.8. Effects of Symbiotic Fungi Reinoculation on Plant Height, Rejuvenation, and Sprouting of Blueberry

**Figure 9:**
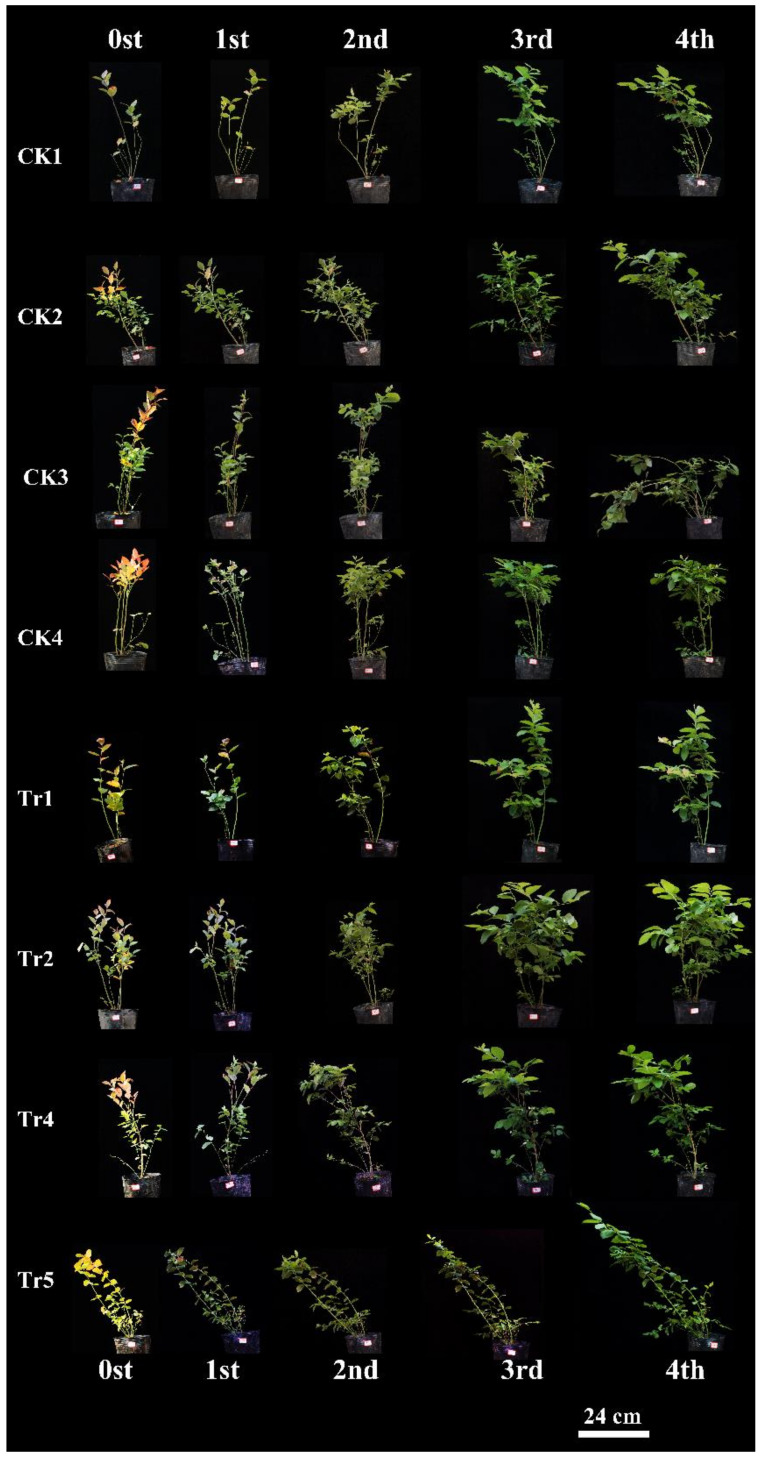
Phenotypes of 3-year-old ‘Star’ blueberry plants during 40 days of growth under normal conditions after reinoculation with *Sistotrema*. Plants in the control group were inoculated with PDA without microorganisms.CK1-5 were controls, Tr1-5 were reinoculated treatments.

**Figure 10:**
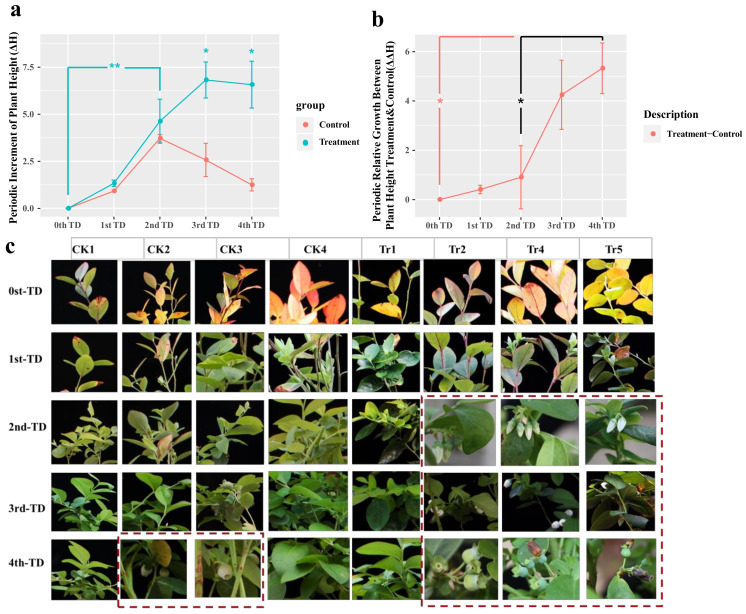
Growth-promoting effects of *Sistotrema*. (**a**) Periodic growth and (**b**) periodic relative growth (plant height) of 3-year-old Star blueberry plants during 40 days of growth after reinoculation with *Sistotrema*. (**c**) Leaf phenotypes of 3-year-old ‘Star’ blueberry plants during 40 days of growth under normal conditions after reinoculation with *Sistotrema*. Asterisks indicate significant differences (*p* < 0.05 *, *p* < 0.01 **). Plants in the control group were inoculated with PDA without microorganisms. CK1-5 were controls, Tr1-5 were reinoculated treatments.

Reinoculation with *Sistotrema* symbiotic fungi significantly affected the growth of 3-year-old ‘Star’ blueberry plants under normal conditions (Figure 9). In each period after reinoculation, the increase in the height of blueberry plants was significantly higher in the treatment group than in the control group, especially in the later stage (3rd TD–4th TD). During the later stage after reinoculation (3rd TD–4th TD), blueberry plant height (Figure 10a) increased by 6.1 (ΔH) in the treatment group, but did not increase in the control group. The difference in periodic relative growth (ΔΔH) between the reinoculated group and the control group became larger over time (*p* < 0.05, Figure 10b). We speculated that this difference may have become even larger after 40 days.

Observations of the changes in various parts of blueberry plants revealed regreening of reinoculated plants, but not control plants, in the first period (1st TD). Flower buds appeared on day 20 (2nd TD) in the reinoculated group, but on day 40 (4th TD) in the control group. Abscission of flowers and fruit occurred in the reinoculation group on day 40 (4th TD) (Figure 10c).

**Figure 11:**
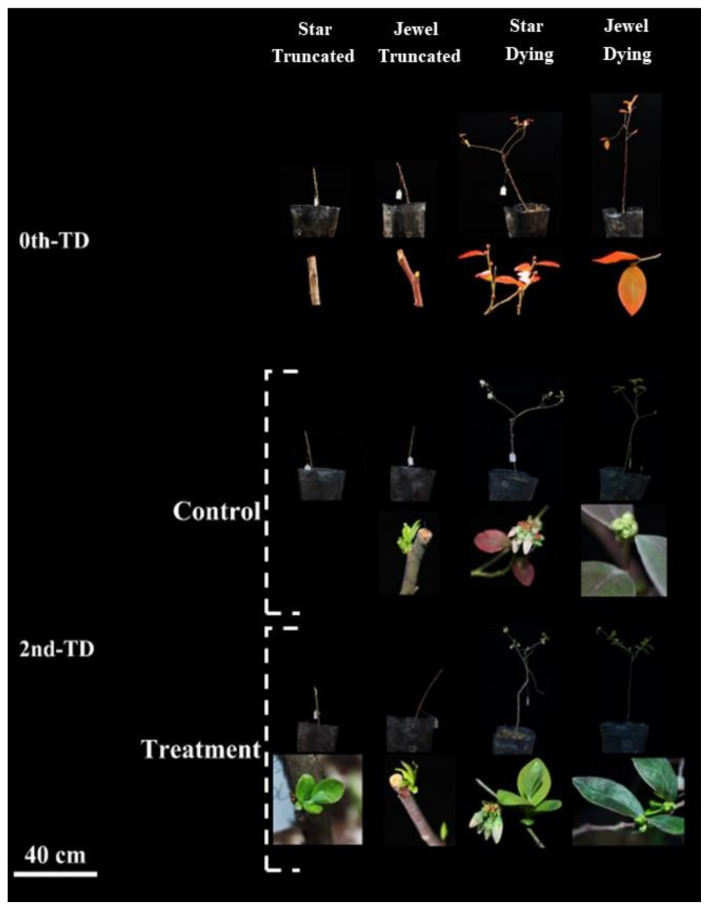
Recovery and sprouting of 3-year-old ‘Star’ and ‘Jewel’ blueberry plants. Plants in the control group were inoculated with PDA without microorganisms.

Reinoculation with *Sistotrema* caused 3-year-old dying plants of the ‘Star’ and ‘Jewel’ blueberry (large-scale leaf loss and leaf yellowing) to recover (Figure 11). Compared with the dying ‘Star’ and ‘Jewel’ plants in the control group, those reinoculated with *Sistotrema* symbiotic fungi showed significantly better growth. For ‘Star’ blueberry plants, those in the control group flowered, but the leaves remained dark red, while those in the group reinoculated with *Sistotrema* showed healthy growth with green leaves. Comparing the two varieties, ‘Jewel’ showed stronger stress resistance and rejuvenation ability compared with ‘Star.’ *Sistotrema* was able to rejuvenate and restore the growth of all the tested blueberry varieties.

Reinoculation with *Sistotrema* symbiotic fungi promoted the budding of 3-year-old stunted ‘Star’ and ‘Jewel’ blueberry plants (Figure 11). At 20 days after inoculation, the stunted ‘Star’ blueberry plants had formed new buds on the stem, but those in the control had not. The ‘Jewel’ blueberry plants formed new buds in the control and reinoculated groups, but the buds were larger in the reinoculated group.

## 4. Discussion

### 4.1. Differences of Fungal Diversity in Rhizosphere Soil under Different Growth States and Different Cultivars of Blueberry

Crop plants, especially cash crops, are often cultivated long-term, so stable soil conditions are particularly important [41]. During long-term cultivation, the root exudates of normal plants affect the microbial composition of the rhizosphere and nonrhizosphere soils—a phenomenon known as the rhizosphere effect. The rhizosphere effect can promote the aggregation and multiplication of some microorganisms or inhibit the survival of others, and these differences become more pronounced with prolonged cultivation time [42]. Our experiments show that blueberry roots can specifically enrich soil fungi, consistent with previous research [43]. In studies on rhizosphere microbes that are specifically enriched by the root exudates of blueberry, the fungal category has great research value (Figure 1, Figure 3b and Figure 4).

Soil microorganisms can decompose and transform humus into nutrients that plants use for growth. Therefore, the diversity of soil microorganisms can often reflect the stability of the soil ecosystem, and this is an indicator of soil health [29]. In this study, among the three blueberry varieties tested, ‘Premier’ blueberry (JG, JB) had stronger roots and the best health, while ‘O’Neal’ blueberry (O) and ‘Sharpblue’ blueberry (XB, XG) were attack by pathogenic bacteria (Figure 1 and Figure 2a,d). The health and diversity of the soil microbial community was highly consistent with the growth and physiological performance of blueberry plants. In production, ‘Premier’ blueberry produces poor-quality fruit, but it has some outstanding physiological advantages such as a robust root system, strong adaptability, and high yield [40], all of which are related to root microbial activity and function. At the same time, ‘Premier’ soils contained the fewest pathogenic fungi (Figure 3d). The results show that, compared with ‘O’Neal’ and ‘Sharpblue,’ ‘Premier’ blueberry showed better growth in terms of biomass, and its root exudates had a stronger effect on the soil microbial environment. Therefore, we speculate that, compared with the other varieties, ‘Premier’ had a stronger effect to select for beneficial symbiotic fungi in the rhizosphere [44].

### 4.2. Identification of Core Symbiotic Fungi Unique to ‘Premier’ Blueberry

During long-term cultivation, the root exudates of 5-year-old ‘Premier’ blueberry support or inhibit soil microorganisms, resulting in a unique soil microenvironment in its rhizosphere [42]. Through the co-occurrence network and symbiosis analyses of ‘Premier’ rhizosphere fungi (Figure 2c and Figure 3a,c,d), six core rhizosphere fungi with putative symbiosis ability were identified. We focused on isolating *Sistotrema*, the most abundant of these fungi.

Soil microorganisms can fix nutrients in soil inside their cells and transform them into forms that can be directly used by plants, thereby increasing nutrient availability to plants. Reinoculation of symbiotic fungi can effectively improve the soil microbial community structure and transform its surroundings to create a suitable soil environment for host plants and promote their growth. At the same time, healthy dominant plants can nourish the reproduction of symbiotic fungi [45]. The COG functional analysis (Figure 5) revealed that *Sistotrema* fungi can effectively fill the shortcomings of weak nutrient absorption capacity of blueberry roots [13]. In this way, they have the potential to increase plant yield and/or growth, especially considering their abundance in rhizosphere soil (Figure 2c). Thus, there is considerable value in isolating *Sistotrema* fungi and using them to reinoculate plant roots.

### 4.3. Growth-Promoting Effect of Sistotrema Reinoculated onto Blueberry

Symbiotic fungi interact with plant roots to promote plant photosynthesis and enhance their stress tolerance. After reinoculation of *Sistotrema* symbiotic fungi, the content of Fe^2+^ (metal cations) and soluble nitrogen in rhizosphere soil were increased (Figure 8a) and the growth was increased obviously (Figure 10). In addition, it can effectively alleviate the degradation of chlorophyll. This growth-promoting effect of *Sistotrema* reinoculation is consistent with the previous studies and the analysis result of COG function [46]. The COG function analysis highlighted several important functions of *Sistotrema*. The first one was the glycoside hydrolase family proteins (G). These proteins degrade substrates such as cellulose, xylan, chitin, and other polysaccharides, thereby increasing the nitrogen content in soil. At the same time, these proteins can degrade other complex substrates in soil, such as lignocellulose or coconut bran, to provide nutrients for plant growth [47]. The ferric reductase (T) function of *Sistotrema* fungi refers to the conversion of Fe complexes into Fe^2+^ [48]. This function, combined with a divalent metal cation uptake system (P), can effectively improve the absorption of Fe^2+^ from soil (Figure 8a). Another important function of *Sistotrema* fungi was the ammonium transporter family/bivalent metal cation uptake system/nitrite and sulfite reductase (P), through which NH_4_^+^ can be taken up from soil by ammonium transporters [49]. In addition, NO_2_^-^ is converted to ammonia by nitrite reductase [50], and this inhibits nitrification–denitrification to reduce nitrogen losses from soil (Figure 8b–d). Ferrous insertion into protoporphyrin IX (H) is involved in chlorophyll synthesis [51], and ubiquitin carboxy–terminal hydrolase (O) completes de-ubiquitination without the participation of ATP to stabilize ubiquitin in cells [52]. This inhibits the degradation of chloroplasts [53] (Figure 6a). Cytochrome P450 (Q) allows fungi to degrade heterologous substances, thereby remediating environmental pollution [54]. Reinoculation with *Sistotrema* also protected the root activity of blueberry plants (Figure 6b), increased their stress resistance, rejuvenation, and budding (Figure 9, Figure 10 and Figure 11), and promoted early flowering and fruiting (Figure 10c). The above results show that *Sistotrema* has a significant effect on promoting growth and verify the feasibility of the strategy of directional screening and isolation of beneficial symbiotic fungi based on functional analyses.

In the early stage after reinoculation, adequate soil nutrients are particularly important for the establishment of the symbiotic relationship between the plant host and the symbiotic fungus. Previous studies have suggested that symbiotic fungi obtain nutrients from the soil for their propagation and infection. If there are insufficient nutrients in the soil, then the symbiotic fungus will take nutrients from plant roots, resulting in plant death [55]. Our results show that *Sistotrema* consumed a large amount of soil nutrients in the first 20 days after reinoculation, and then gradually released the fixed nutrients to blueberry plants with the establishment of the symbiotic relationship 20 days later. This also explains why there was no difference in the relative change in blueberry plant height (ΔΔH) between the control and the control before 20 days (Figure 10b).

## 5. Conclusions

This study is the first to report the directional separation and reinoculation of *Sistotrema* symbiotic fungi and its growth-promoting effect. Our study also demonstrates the use of fungi genomic function analysis for the directional screening and isolation of beneficial symbiotic fungi. Our results suggest that reinoculation of rhizosphere soil microorganisms can improve the survival rate and yield of most blueberry varieties. These findings lay the foundation for the further development of microbial fertilizers specifically formulated for blueberry plants.

## Figures and Tables

**Table 1:** Details of soil samples collected in this study.

Blueberry Variety	Growth State	Plant Character	Sampling Site	Sample Code
‘O’Neal’	Weak growth	Flourishing	Rhizosphere	O
		Flourishing	Nonrhizosphere	OK
‘Sharpblue’	Medium growth	Flourishing	Rhizosphere	XG
		Flourishing	Nonrhizosphere	XGK
		Weak	Rhizosphere	XB
		Weak	Nonrhizosphere	XBK
‘Premier’	Strong growth	Flourishing	Rhizosphere	JG
		Flourishing	Nonrhizosphere	JGK
		Weak	Rhizosphere	JB
		Weak	Nonrhizosphere	JBK

**Table 2:** Characteristics of blueberry materials used in reinoculation tests.

Blueberry Variety	Classification	Plant Character	Treatment
1-year-old ‘Gulfcoast’	Southern highbush blueberry	Normal growth	Drought and nutrient-deficiency stress
3-year-old ‘Star’	Southern highbush blueberry	Normal growth	Normal treatment
		Stunted	Normal treatment
		Dying	Normal treatment
3-year-old ‘Jewel’	Southern highbush blueberry	Stunted	Normal treatment
		Dying	Normal treatment

## Data Availability

Not applicable.

## Supplementary Materials

The following supporting information can be downloaded at: https://www.mdpi.com/article/10.3390/jof9070779/s1, Table S1: Constituents of 10 × Hoagland’s nutrient solution for soil; Table S2: Sequences of primers used to amplify ITS1 sequences; Table S3: Primers for PCR screening of re-inoculation fungi; Table S4: Constituents of DNS reaction solution; Table S5: The 30 symbiotic fungi in the blue community.
