# Supplementary material for: A Symbiotic Fungus Sistotrema Benefits Blueberry Rejuvenation and Abiotic Stress Tolerance
Source: J Fungi (Basel). 2023 Jul 24;9(7):779. doi: 10.3390/jof9070779 (PMC10381331; doi:10.3390/jof9070779)
Supplement: Supplementary file 1 [file jof-09-00779-s001.zip › jof-2398397-supplementary.pdf]

Table S1 Constituents of 10 × Hoagland's nutrient solution for soil

Composition	Concentration (g/L)
Ca $(NO_3)_2 \cdot 4H_2O$	9.45
KNO3	5.06
NH4NO3	0.80
KH2PO4	1.36
MgSO4	4.93
FeSO4·7H2O	5.56
EDTA-2Na	7.46

Table S2 Sequences of primers used to amplify ITS1 sequences

Primer name	Sequences（5’→3’）	Gene length
	ITS1	
ITS1F	TCCGTAGGTGAACCTGCGG	388-583 bp
ITS2R	TCCTCCGCTTATTGATATGC	

Table S3 Primers for PCR screening of re-inoculation fungi

Primer name	Sequences（5’→3’）	Gene length
Ag-Ca F	CAACTTTTAACAACG	150-200 bp
Ag-Ca R	CACTCGAACAGGCGT	

Table S4 Constituents of DNS reaction solution

composition	Concentration(g/L)
DNS	6.50
NaOH	26.00
Glycerol	45.00

Table S5 The 30 symbiotic fungi in the blue community

Number	Name
1	o_Cantharellales; f_Cantharellales_fam_Incertae_sedis; g_*Sistotrema*
2	o_Helotiales; g_*Spirosphaera*
3	g_*Calcarisporiella*
4	o_ Pleosporales; f_Didymellaceae; g_*Boeremia*
5	o_Moniliales; f_Dematiaceae; g_*Coniosporium*
6	o_ Orbiliales; g_*Microdochium*
7	o_ Saccharomycetales; g_*Candida*
8	o_ Hypocreales; f_Clavicipitaceae; g_*Pochonia*
9	o_Pleosporales; f_Cucurbitariaceae; g_*Pyrenochaetopsis*
10	o_Pezizales; f_Ascobolaceae; g_*Ascobolus*
11	o_Teloschistales; f_Teloschistaceae; g_*Athallia*
12	o_Sordariales; f_Chaetomiaceae; g_*Corynascella*
13	o_Pleosporales; f_Latoruaceae; g_*Latorua*
14	o_Eurotiales; f_Trichocomaceae; g_*Sagenomella*
15	f_Orbiliaceae; g_*Dactylella*
16	g_*Guehomyces*
17	f_Orbiliaceae; g_*Duddingtonia*
18	o_Archaeorhizomycetales; f_Archaeorhizomycetaceae; *g_Archaeorhizomyces*
19	o_Tremellales; f_Trimorphomycetaceae; *g_Saitozyma*
20	o_Pleosporales; f_Tetraplosphaeriaceae; g_*Tetraplosphaeria*
21	o_Cystobasidiales; f_Microsporomycetaceae; g_*Microsporomyces*
22	o_Agaricales; f_Strophariaceae; g_*Deconica*
23	o_Malasseziales; f_Malasseziaceae; g_*Malassezia*
24	o_Russulales; f_Stephanosporaceae; g_*Unidentified*
25	o_Cantharellales; f_Ceratobasidiaceae; g_*Unidentified*
26	o_Hymenoptera; f_Orbiliaceae; g_*Unidentified*
27	o_Sordariales; f_Chaetomiaceae; g_*Unidentified*
28	o_Hypocreales; f_Clavicipitaceae; g_*Unidentified*
29	o_Pezizales; f_Pyronemataceae; g_*Unidentified*
30	o_Pleosporales; f_Unidentified; g_*Unidentified*
